# Unexplained Aspects of Anemia of Inflammation

**DOI:** 10.1155/2010/508739

**Published:** 2010-03-24

**Authors:** Elizabeth A. Price, Stanley L. Schrier

**Affiliations:** ^1^Department of Medicine (Hematology), Stanford University, Stanford, CA 94305-3434, USA; ^2^Division of Hematology, Stanford Comprehensive Cancer Center, 875 Blake Wilbur Drive, Stanford, CA 94305, USA

## Abstract

Anemia of inflammation (AI), also known as anemia of chronic inflammation or anemia of chronic disease was described over 50 years ago as anemia in association with clinically overt inflammatory disease, and the findings of low plasma iron, decreased bone marrow sideroblasts and increased reticuloendothelial iron. Pathogenic features underlying AI include a mild shortening of red cell survival, impaired erythropoietin production, blunted responsiveness of the marrow to erythropoietin, and impaired iron metabolism mediated by inflammatory cytokines and the iron regulatory peptide, hepcidin. Despite marked recent advances in understanding AI, gaps remain, including understanding of the pathogenesis of AI associated with “noninflammatory” or mildly inflammatory diseases, the challenge of excluding iron deficiency anemia in the context of concomitant inflammation, and understanding more precisely the contributory role of hepcidin in the development of AI in human inflammatory diseases.

## 1. Introduction

Anemia of inflammation (AI), also known as anemia of chronic inflammation or anemia of chronic disease, was described over 50 years ago as a normo to microcytic anemia, typically of mild severity, characterized biochemically by a low plasma iron, decreased total iron-binding capacity, decreased transferrin saturation, and, on bone marrow examination, decreased sideroblasts and increased reticuloendothelial iron [1]. Despite the heterogeneity of underlying infectious, malignant and inflammatory diseases described in conjunction with this type of anemia, there was a remarkable degree of similarity in terms of the severity of the anemia, correlating directly with the degree of inflammation present, and its stability over time, features which remain characteristic of AI today [1]. Despite advances in understanding the underlying mechanisms leading to AI, several key clinical issues remain, including understanding of mechanisms of AI in “noninflammatory” diseases, optimal methods for distinguishing AI from iron deficiency anemia, and understanding the contributory role of various pathologic mechanisms in individual human diseases that lead to AI. 

## 2. Pathophysiology of AI

Pathogenic features underlying AI include a mild shortening of red cell survival, impaired erythropoietin production, blunted responsiveness of the marrow to erythropoietin, and impaired iron metabolism [2]. Inflammatory cytokines, including interleukin-1 (IL-1), tumor necrosis factor-*α* (TNF-*α*), and interferon-*γ* (IFN-*γ*), affect the differentiation and proliferation of erythroid progenitor colony formation in vitro [2–4]; the proapoptotic effects of which have been reversed by treatment with the TNF-inhibitor infliximab in patients with rheumatoid arthritis [5, 6]. Cytokines and acute-phase reactants may also cause sequestration of iron in reticulo-endothelial cells (*reviewed in *[4]); however, hepcidin, a key regulator of iron homeostasis, has recently emerged as a central player in the disordered iron metabolism seen in AI. The hepcidin gene is located on chromosome 19 and encodes an 84 aa prepropeptide which undergoes cleavage to produce first the circulating 60 aa prohepcidin, followed by cleavage to produce the 25 aa form of hepcidin [7]. This 25 aa peptide acts via binding to the only known mammalian cellular iron exporter, ferroportin, leading to its internalization and degradation, with consequent loss of iron export from the duodenal enterocytes and reticulo-endothelial cells [8]. Hepcidin synthesis is suppressed by erythropoiesis [9] and iron deficiency and upregulated by iron overload and inflammation. Infusion of IL-6 [10] or lipopolysaccharide (LPS) [11] into healthy volunteers leads to a rapid increase in urinary hepcidin, with concomitant decrease in serum iron levels. IL-6 upregulates synthesis of hepcidin via signal transducer and activator of transcription-3 (STAT-3) signaling [12]. Hepcidin levels have been found to be elevated in patients with severe inflammation when compared to healthy controls [13]. A hepcidin transgenic mouse model replicates many of the pathogenic features of AI, including a mild, hypochromic anemia, sequestration of iron in the spleen, and impaired marrow response to erythropoietin [14]. Thus, in vitro and in animal and human models, hepcidin has been shown to effect most if not all of the iron-related changes which are pathognomic features of AI. 

## 3. AI in “Noninflammatory” States

Diagnostic features of AI in the appropriate clinical context include characteristic iron indices associated with reduced bone marrow sideroblasts and increased reticuloendothelial iron [1]. In current practice, the diagnostic criteria of AI are frequently modified to require only the presence of anemia, the absence of overt other etiologies, and associated peripheral blood findings of disordered iron metabolism, such as a low serum iron with normal or elevated serum ferritin [15]. This has led to widening of the spectrum of clinical states associated with AI, including aging, diseases associated with aging such as heart failure, and hospitalization [16]. It is important to note, however, that there are currently no uniform peripheral blood laboratory criteria for the diagnosis of AI. 

### 3.1. Anemia of the Elderly

Abnormal iron indices suggestive of AI are relatively common findings in elderly patients. In the U.S. National NHANES III study, 20% of elderly adults with anemia were characterized as having AI as the sole cause of their anemia, defined as a serum iron <60 mcg/dL and no evidence of iron deficiency. Iron deficiency was in turn defined as having at least two of the following: transferrin saturation less than 15%, serum ferritin less than 12 ng/mL, or erythrocyte protoporphyrin concentration greater than 1.24 uM [17]. Aging is also associated with increased inflammation and a mild rise in inflammatory markers, including IL-6 [18, 19], and elderly patients with anemia have been found to have higher levels of inflammatory markers than elderly patients without anemia [20]. However, the degree of inflammation present is mild; in one study the differences in median levels of IL-6 and C-reactive protein (CRP) between anemic and nonanemic elderly patients were relatively small (2.5 versus 1.4 pg/mL and 4.9 versus 2.7 mg/L, resp.) [21]. 

Can AI-type pathophysiology exist in the absence of associated clinically evident inflammatory disease? This question has been studied in elderly patients. In an early report, 5 elderly patients without underlying inflammatory disease presented with a microcytic, hypochromic anemia, low serum iron levels, and increased bone marrow iron [22]. In ferrokinetic studies, these patients were found to have low iron absorption, normal iron utilization (incorporation of infused iron into red cells), and decreased reutilization of iron (incorporation of iron from infused hemoglobin into red cells). These findings of low serum iron, low iron binding capacity, high serum ferritin, and increased bone marrow iron in elderly patients in the absence of malignant or inflammatory diseases were termed “primary defective iron-reutilization syndrome” [23]. Interestingly, treatment with testosterone enanthate led to improvements in hemoglobin, red cell mass, and iron reutilization. In a follow-up study, two similar patients were described [23]. Treatment with danazol in both patients resulted in an increases in hemoglobin, serum iron, percent transferrin saturation, and decreased ferritin, suggesting that treatment led to release of previously sequestered reticulo-endothelial iron. These results are intriguing and suggest both that disordered iron metabolism can develop in the absence of overt inflammatory disease, and also that androgen therapy, at least in this small patient sample, corrected the underlying defect. Androgens have been shown to increase erythropoietin levels [24] and hematopoietic stem cell cycling [25]; however it seems more likely that an unrelated mechanism led to improvement in the anemia and iron homeostasis in these cases. It would also be interesting to assess whether milder biochemical evidence of disordered iron metabolism, that is, a mild normocytic normochromic anemia with less dramatically abnormal iron indices, had similar ferrokinetic findings. It is possible that the use of more sensitive markers of inflammation such as the high sensitivity CRP would isolate a subset of patients without overt inflammatory disease who nonetheless had findings consistent with AI.

### 3.2. Anemia of Heart Failure

AI as defined by iron indices is also common in patients with congestive heart failure. In one study, 85 of 148 (57%) anemic outpatients with congestive heart failure had AI [26]. Those with AI had higher levels of inflammatory markers, including IL-6 and TNF-*α*, compared to levels found in previously reported healthy subjects, supporting the diagnostic criteria. However, in another study in anemic hospitalized patients with more advanced heart failure, 27 of 37 (73%) had iron deficiency as diagnosed by the gold standard of absent iron on bone marrow aspirate, despite the group having a mean ferritin of 75 ± 59.1 ng/mL [27]. Thus, using a serum ferritin cutoff level of >50 ng/mL may be too restrictive in excluding iron deficiency anemia in patients with heart failure. The findings from this study highlight the dilemma of distinguishing iron deficiency anemia from AI, which remains a frequent clinical challenge despite the availability of tests such as the soluble transferrin receptor (sTfR), a marker of increased erythropoiesis and iron deficiency [28].

### 3.3. Identifying Iron Deficiency in Patients with AI

Recent work suggests that measuring hepcidin levels may have future utility in distinguishing the two disorders. In a rat model of chronic inflammation and iron deficiency anemia, hepatic hepcidin mRNA was suppressed in controls with iron deficiency alone, elevated in rats with inflammation alone, and suppressed in rats with combined inflammation and iron deficiency [29]. Serum hepcidin levels followed a similar but less distinct pattern. Rats with inflammation had significantly less uptake of orally administered iron compared with both control rats and rats with combined inflammation and iron deficiency. In the same study, human patients were designated as having anemia of inflammation in combination with iron deficiency if they had anemia, an associated inflammatory disease, and a transferrin saturation <16%, serum ferritin of <100 mcg/mL and sTfR/log ferritin >2. Serum hepcidin levels were significantly higher in patients with inflammation alone than in those with either iron deficiency alone or combined iron deficiency and inflammation. Consistent with these results, in human duodenal biopsy samples, mRNA expression of ferroportin was increased in those with combined inflammation and iron deficiency compared to those with inflammation alone. In another study utilizing a mouse model of sepsis and prolonged organ dysfunction, inflammation-induced increase in hepatic hepcidin mRNA was suppressed by phlebotomy, and, to a lesser extent, by erythropoietin administration [30]. These results suggest that severe iron deficiency exerts a dominant effect over inflammation with regard to regulation of hepcidin in vivo, and furthermore, that hepcidin levels, if validated for clinical use, may be of utility in confirming or excluding iron deficiency in the presence of concomitant inflammation. 

### 3.4. Anemia of Chronic Kidney Disease (CKD)

Anemia of CKD is another “noninflammatory” disease in which inflammation may actually play a prominent role in the development of anemia. The pathogenesis of anemia in CKD is multifactorial and predominately driven by impaired erythropoietin production; however inflammation negatively impacts erythropoiesis and is associated with resistance to erythropoietic therapy [31, 32]. Several recent studies have measured hepcidin levels in patients with CKD in an effort to clarify the contributory role of hepcidin and disordered iron metabolism in anemia in this disease. Ashby et al. measured plasma hepcidin (by immunoassay) in healthy controls and patients with chronic kidney disease both off (44 patients) and on (94 patients) hemodialysis [33]. Although hepcidin levels did not correlate strongly with ferritin levels in patients on hemodialysis (felt by the authors to be related to intravenous iron supplementation in this group), in those not on hemodialysis, hepcidin levels did correlate strongly with ferritin levels, consistent with upregulation of hepcidin in relation to increased iron. In addition, hepcidin levels were negatively correlated with erythropoietin dose, and fell slightly in patients who were initiated on erythropoietin therapy. There was no correlation between hepcidin levels and markers of inflammation (CRP or IL-6) in either group. Interestingly, there was a negative correlation between hepcidin and worsening renal function. Plasma growth differentiation factor-15 (GDF15) levels, which are elevated in thalassemia major and inhibit hepdcidin synthesis [34], did not appear to negatively regulate hepcidin in this setting, consistent with results in normal volunteers treated with low-dose recombinant human erythropoietin [35]. Weiss et al. measured serum hepcidin as well as iron indices in 20 patients on hemodialysis and found that hepcidin levels correlated positively with serum iron, ferritin, transferrin saturation, and negatively with reticulocyte count, but not with markers of inflammation, including serum IL-6, transforming growth factor-*β* (TGF-*β*), or CRP [36]. However, the patients had relatively low levels of inflammation, with mean CRP levels of only 0.7 mg/dL. Similarly, Tomosugi et al. measured hepcidin levels using a semiquantitative surface-enhanced laser desorption ionization time of flight mass spectrometry (SELDI-TOF MS) assay and found that while serum hepcidin-25 levels correlated well with IL-6 levels in patients with acute infection, there was no correlation between hepcidin and IL-6 in patients receiving dialysis without additional associated inflammatory disease [37]. Zaritsky et al. found in adults with chronic kidney disease stages 2 to 4, ferritin, soluble transferrin receptor levels and glomerular filtration rate (GFR) were directly correlated with serum hepcidin levels. When pediatric patients with CKD stages 2–5 were included in a multivariate analysis, high resolution CRP also correlated with serum hepcidin levels [38]. Thus, these studies, taken together, suggest that in patients with CKD without additional inflammatory disease, hepcidin is regulated predominately by iron status, and to a lesser extent by erythropoiesis, and even less by inflammation and thus may not play a prominent contributory role in the anemia associated with CKD.

## 4. Hepcidin Levels in Inflammatory Bowel Disease

Measurements of hepcidin levels in inflammatory bowel disease (IBD) have also yielded interesting results. In one study, six pediatric patients with active Crohn's disease (defined as an IL-6 level >5 pg/mL) had poor absorption of a single dose of oral iron and high urinary hepcidin levels (mean 295.3 ng/mg creatinine) compared to ten patients with relatively inactive Crohn's disease (with IL-6 levels ≤5 pg/mL and mean urinary hepcidin levels 31.22 ng/mg creatinine). Thus, patients with active disease had a markedly higher hepcidin levels than those with quiescent disease, consistent with the hepcidin model of AI. Interestingly, hemoglobin levels were similar in both groups (mean 11.7 and 11.6 g/dL in active and inactive disease, resp.). Although this was possibly due to the small number of patients studied, the absence of a difference in hemoglobin level despite markedly different hepcidin levels also raises the possibility that elevated hepcidin does not drive the anemia in this disease, even when active. Surprisingly, in another study in 61 patients with inflammatory bowel disease (mean age 44 years), serum hepcidin levels, as measured by radioimmunoassay, were significantly lower in those with IBD compared to healthy controls. In this study, iron deficiency anemia was defined as the presence of a low serum iron (<10.6 umol/L) and low serum ferritin (<15 ug/L). AI was defined as anemia with low serum iron and normal ferritin level. Although the directly contrasting results between these two studies may potentially be explained by differences in urine versus serum hepcidin assays, an alternative explanation is that patients with IBD with a serum ferritin above 15 may still be iron deficient and have suppressed hepcidin synthesis. Regardless, these contrasting results highlight the need for further studies to more accurately delineate the contributory role of hepcidin in the development of AI in IBD. 

In summary, despite marked recent advances in understanding AI, gaps remain, including understanding the pathogenesis of AI associated with “noninflammatory” or mildly inflammatory diseases, the challenge of excluding iron deficiency anemia in the context of concomitant inflammation, and understanding more precisely the contributory role of hepcidin in the development of AI in human inflammatory diseases. 
